# Facial skin and soft tissue infection caused by *Mycobacterium wolinskyi* associated with cosmetic procedures

**DOI:** 10.1186/1471-2334-13-479

**Published:** 2013-10-16

**Authors:** Seung Jin Yoo, Keun Hwa Lee, Sung-No Jung, Sang Taek Heo

**Affiliations:** 1 Jeju National University School of Medicine, Jeju-si, Jeju, South Korea; 2 Department of Microbiology and Immunology, Jeju National University School of Medicine, Jeju-si, Jeju, South Korea; 3 Department of Plastic and Reconstructive Surgery, Uijeongbu St. Mary’s Hospital, College of Medicine, The Catholic University of Korea, Seoul, South Korea; 4 Department of Infectious Disease, Jeju National University School of Medicine, Jeju-si, Jeju, South Korea

**Keywords:** *Mycobacterium wolinskyi*, Cosmetic, Filler injection, Skin and soft tissue infection

## Abstract

**Background:**

*Mycobacteirum wolinskyi* is a member of the *Mycobacterium smegmatis* group, which is less frequently found in clinical settings than other nontuberculous mycobacterium (NTM) species. However, its clinical significance has recently increased in opportunistic infections. This case is the first report of facial skin and soft tissue infection by *M. wolinskyi* complicating cosmetic procedures.

**Case presentation:**

A 56-year-old Asian female patient with a history of receiving multiple facial cosmetic procedures over the preceding 2 years was admitted to our institution with swelling, local pain, and erythema on the right cheek. *Mycobacteirum fortuitum* complex isolated from a pus culture was identified as *M. wolinskyi* by *rpoB* sequencing. Metallic foreign bodies and abscess were detected by radiologic imaging. The pus was incised and drained. Treatment comprised clarithromycin (500 mg every 12 h), amikacin (200 mg every 8 h), and ciprofloxacin (400 mg every 6 h).

**Conclusion:**

We report the first case of facial skin and soft tissue infection with *M. wolinskyi* after multiple cosmetic procedures of filler injection and laser lipolysis. Increased occurrence of NTM infection in nosocomial settings suggests the importance of appropriate treatment including culturing and *rpoB* gene sequencing when patients who have undergone cosmetic procedures display symptoms and signs of soft tissue infection indicative of NTM infection.

## Background

Among more than 140 species of nontuberculous mycobacteria (NTM), the rapidly growing mycobacteria (RGM), which are widely distributed in soil and water, is a clinically significant pathogen that causes various human diseases [1]. RGM, a type IV mycobacteria under the Runyon classification system, includes the *Mycobacterium fortuitum* group*, Mycobacterium perginum, Mycobacterium chelonae, Mycobacterium abscessus, Mycobacterium immunogenum, and Mycobacterium smegmatis* group [2]. *Mycobacterium wolinskyi*, which belongs to the *M. smegmatis* group, was first identified in 1999 by 16S rRNA sequencing [3]. *M. wolinskyi* is less frequently observed in clinical settings, but it is predominantly associated with skin and soft tissue infections (SSTIs) [4]. Previously reported cases of *M. wolinskyi* have described its primary association with posttraumatic or postsurgical wounds resulting in cellulitis, osteomyelitis, and localized abscess [5]. Herein, we report the first case of facial SSTI caused by *M. wolinskyi* after multiple cosmetic procedures involving filler injections and laser therapies.

## Case presentation

**Figure 1:**
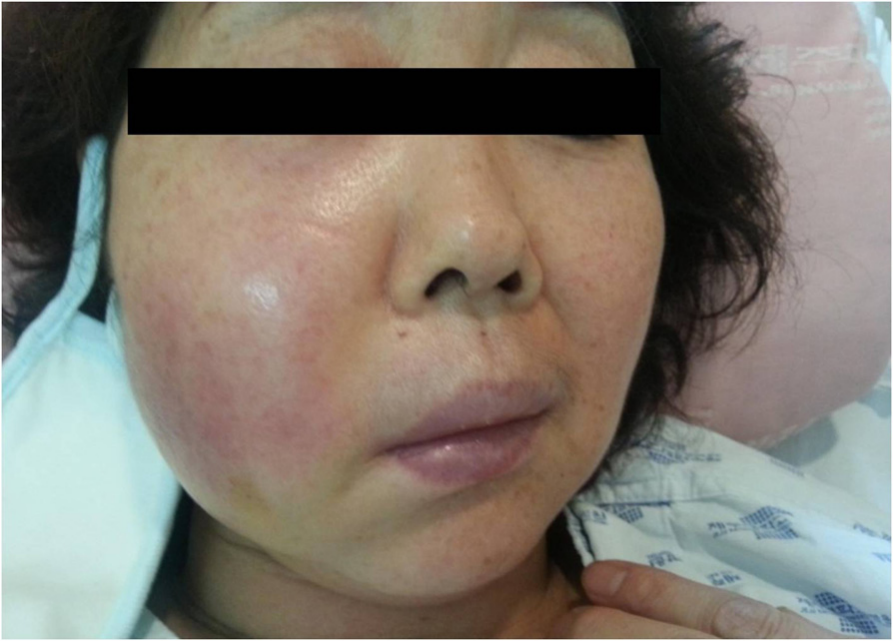
Small indurated nodules with persisting edema on the right cheek.

A 56-year-old Asian female patient was admitted to Jeju National University Hospital with persisting edema that evolved from a small indurated nodule on the right cheek over the course of 3 months (Figure 1). The patient had received multiple AccuSculpt™ laser procedures (1444 mm Nd:YAG) for facial pigmentation removal and lipolysis, and repeated filler injections for cosmetic purposes at a local clinic since 2011. In August 2012, a subcutaneous indurated nodule approximately 1 to 2 cm in diameter developed on the right side of the face. She was injected with hyaluronidase and triamcinolone weekly for subcutaneous nodule. However, swelling at the site of erythema progressively developed. Although a local physician prescribed antibiotics with suspected facial cellulitis, the patient’s condition did not improve.

The patient did not have any previous surgical and medical history of tuberculosis or diabetes mellitus, but was controlled for hypertension. Physical examination indicated stable vital status. The right facial lesion was generally edematous without a definitely elevated margin in addition to a heating sensation and tenderness at the site of swelling (Figure 1). Initial laboratory findings did not indicate any abnormal results other than elevated C-reactive protein (CRP) 7.13 mg/dL and erythrocyte sedimentation rate (ESR) of 69 mm/h. Human-immunodeficiency virus was negative. Chest radiography revealed no apparent active lesions.

**Figure 2:**
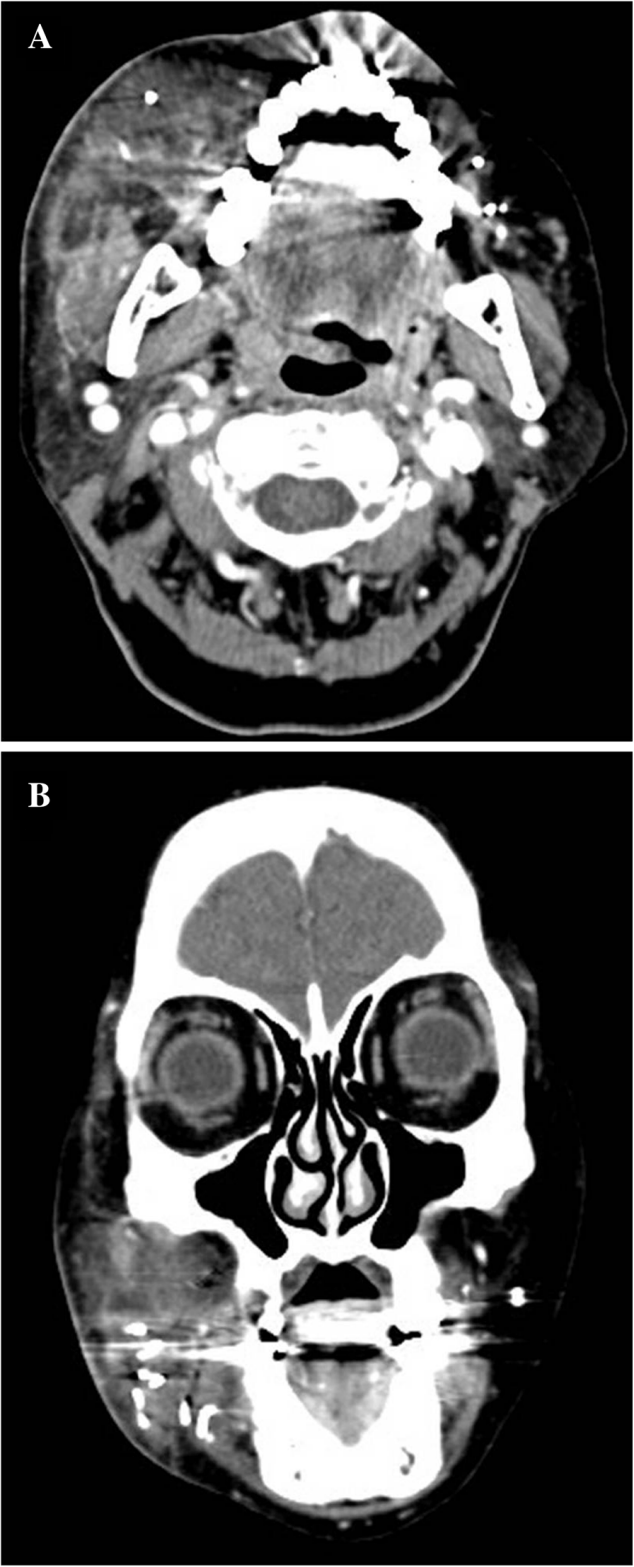
**Facial computed tomography. A**: shows swelling of soft tissue with fatty infiltration on the right side on the axial image **B**: scattered longitudinal tube-like shaped lesions of 0.5 to 1 cm with high density at right mandibular area on the coronal image as well as at the contralateral side.

Facial computed tomography (CT) indicated multiple metallic foreign bodies, soft tissue infection, and fatty infiltration (Figure 2). Since the patient had a history of cosmetic procedures, and steroid injections and showed no apparent improvement of symptoms in response to *β*-lactam antibiotic, pus was collected for acid fast bacilli (AFB) stain, mycobacterium culture, *Mycobacterium tuberculosis*/NTM polymerase chain reaction (TB/NTM PCR), gram staining, and culture. The results indicated the presence of AFB positive and NTM PCR positive organisms. According to the test results, the antibiotic treatment regimen was changed to clarithromycin (500 mg every 12 h), amikacin (200 mg every 8 h), and ciprofloxacin (400 mg every 6 h), and NTM culture for pus was performed. Pain at the site of the lesion was improved, but the patient still complained of continuous pus formation. Meanwhile, *M. fortuitum* complex (MFC) was isolated from the NTM pus culture. The results from the antibiotic susceptibility tests are shown in Table 1. The subsequent *rpoB* gene sequencing identified *M. wolinskyi* with an accuracy of  99%.

**Table 1:** **Antibiotics sensitivity test results for *Mycobacterium wolinskyi***

**Antibiotic**	**Concentration**	**Minimum inhibitory concentration**	**Report**
	**( *μ *g/mL)**	**( *μ *g/mL)**	
Amikacin	1 ~ 128	8	S
Cefoxitin	2 ~ 256	64	I
Ciplofloxacin	0.125 ~ 16	≤ 1	S
Clarithromycin	0.5 ~ 64	4 ~ 16	IR
Doxycycline	0.25 ~ 32	2	S
Imipenam	0.5 ~ 64	16	I
Moxifloxacin	0.125 ~ 16	≤ 0.25	S
Trimethoprimesulfamethoxazole	0.25/4.75 ~ 32/608	16/304	R
Linezolid	2 ~ 64	8	S

S: susceptible/I: Intermediate susceptibility/R: Resistant/IR: Inducible Resistant.

**Figure 3:**
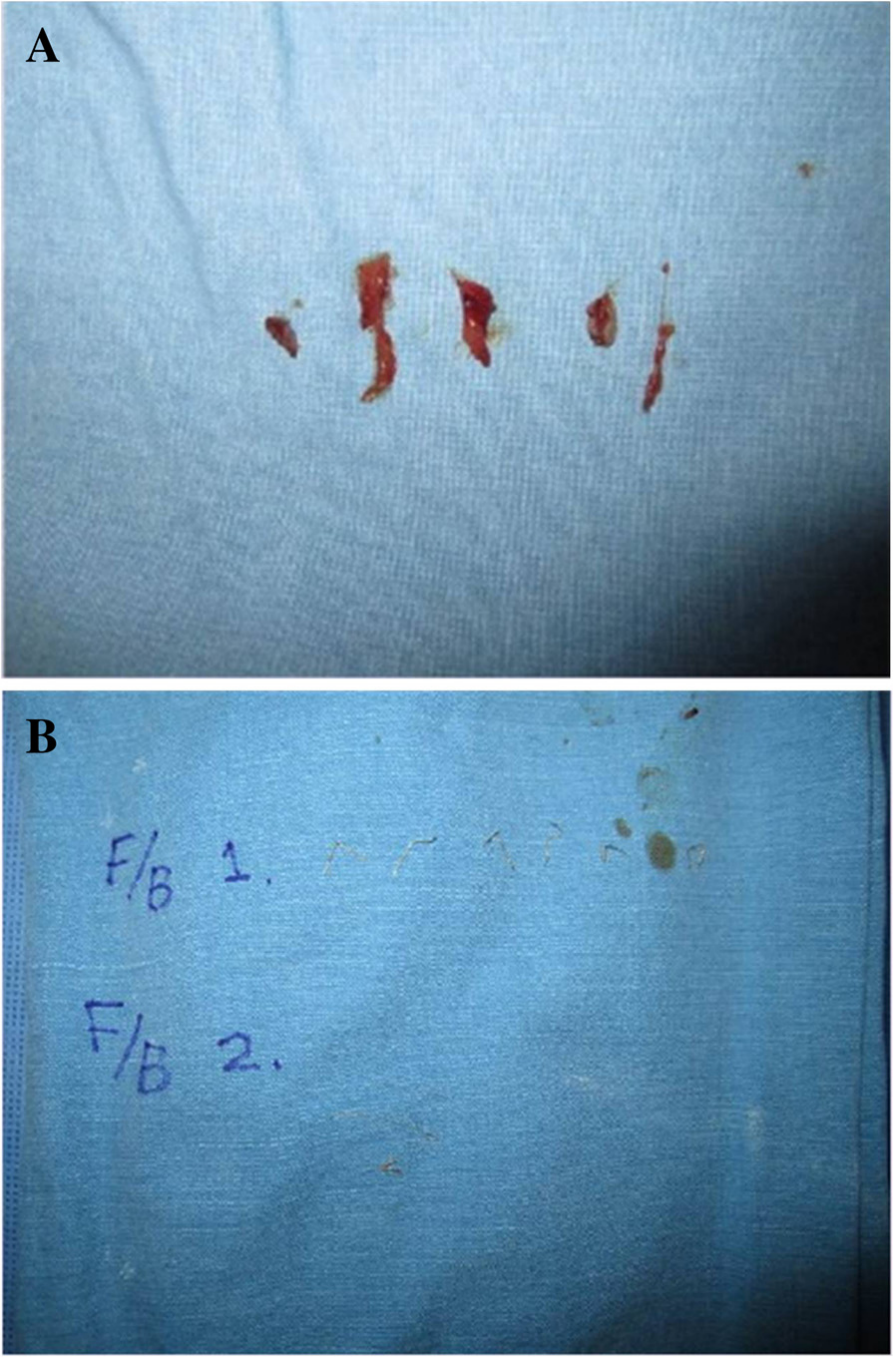
**Postoperative images after incision and drainage. A**: granulation tissue and **B**: Metal foreign body (F/B 1). Remnant thread of previous cosmetic procedure (F/B 2).

**Figure 4:**
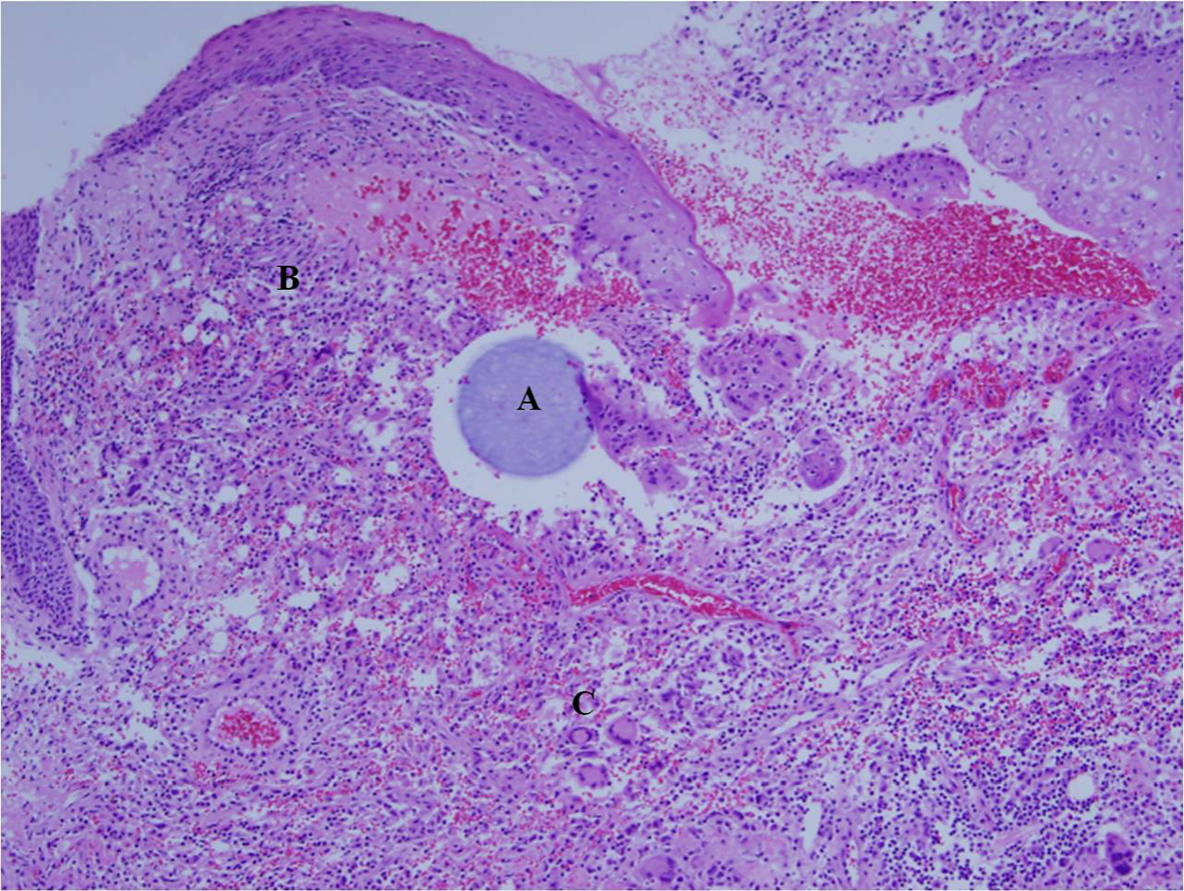
**Postoperative histology findings. A**: Foreign body in dermis; **B**: Lymphocytic infiltration surrounding the foreign body; and **C**: Granulomatous inflammation with multinucleated giant cells (H&E stain, magnification × 100).

Since metal foreign body and abscess had been confirmed by radiologic and pathologic images, incision and drainage was performed to eliminate granulation tissue, metallic foreign bodies, and thread remnants (Figures 3 and 4). At the same time, the patient was treated with oral doxycycline (100 mg every 12 h) and ciprofloxacin (750 mg every 4 h) for 5 months. Subsequently, the facial abscess and erythematous swelling were resolved with minor dermatologic sequelae.

## Conclusions

*M. wolinskyi* has been reported in only 13 cases since its first categorization within the *M. smegmatis* group in 1999. Contrary to several reported cases of SSTI caused by other NTM species [6-10], previously reported cases of *M. wonlinskyi*-related SSTI mostly described incidences following surgical procedures including hip prosthesis, peritoneal dialysis, transplantation, and heart surgery and posttraumatic events [3,11-13]. Infections have also been observed in immunocompromised patients [14,15] and in some cases of tissue necrosis involving subjacent bone [1]. However, *M. wolinskyi* infection complicating facial cosmetic procedure has not hitherto been reported, although there are increasing reports of SSTI caused by other RGM species involving cosmetic therapy such as *M. fortuitum, M. abscessus, M. jacuzzi* and *M*. *concepcionense* (Table 2).

**Table 2:** Summary data of facial skin and soft tissue infection by rapidly growing mycobacterium after cosmetic procedures in the literature

**Case**	**Sex/Age**	**Type of procedure**	**Site**	**Microbiologic finding**	**Reference**
1	F/67	Autologous fat graft	Both cheek	*M. abscessus*	[4]
2	F/50	Autologous fat graft	Both cheek	*M. conceptionense*	[4]
3	F/50	Filler injection	Glabellar area	*M. fortuitum*	[4]
4	M/66	Autologous fat graft	Forehead	*M. abscessus*	[4]
5	F/50	Liposuction and lipoinjection	Both cheek	*M. conceptionenese*	[6]
6	F/50	Filler injection	Right cheek	*M. chelonae*	[8]
7	F/56	Filler injection and accusculpt lipolysis	Right cheek	*M.wolinskyi*	Current case

M: male; F: female.

In this case, we describe a facial SSTI associated with *M. wolinskyi*, presumably due to invasive cosmetic procedures. The exact route and time of the infection is currently unclear because the patient had received multiple cosmetic procedures involving filler injection, and lipolysis over the course of 2 years. One possible route of infection includes a failure to maintain aseptic procedure during the invasive procedures. Furthermore, the patient also has a history of receiving facial acupuncture procedures in the childhood. Multiple acupuncture needle insertions tend to leave needle remnants at the site of procedures, and would allow *M. wolinskyi* to invade dermis and grow along the existing metal fragments more rapidly than without any foreign bodies. Facial acupuncture is a widely-performed childhood ritual on Jeju Island for health and longevity.

Culture and laboratory identification of NTM can be a time consuming process because the process relies on growth rate, pigmentation, and several biochemical tests [1]. Molecular analysis using 16S rRNA and *rpoB* gene sequencing and TB/NTM PCR screening has made the identification process more rapid and accurate [4,5]. In the current case, since PCR and culture results indicated the presence of NTM, *rpoB* gene sequencing analysis was performed using primers MF (5′-CGACCACTTCGGCAACCG-3′) and MR (5′-TCGATCGGGCACATCCGG-3′) [12]. The isolate was identified as *M. wolinskyi*, whose sequence similarity was 99% with *M. wolinskyi* ATCC 700010. Since the *rpoB* gene sequence is more divergent and discriminable for identification of RGM species than 16S rRNA sequence [1], we only performed *rpoB* sequence analysis for prompt identification of NTM species to prevent further delay in diagnosis and treatment in this case.

RGM organisms are generally known to be resistant to standard anti-tuberculosis agents, and treatment for each RGM species differs depending on the susceptibility to antibiotics. In this case, the patient had frequent exposure to medical procedure-related contamination, and the SSTI did not improve with antibiotics and remained for a long duration. Due to its chronic condition and procedure-related infection, not only bacterial but also NTM infections were suspicious. Since Korea is a known tuberculosis endemic area, when infection persists and is resistant to standard antibiotic treatment, tuberculosis infection is more likely than NTM infection. However, failure of standard anti-tuberculosis agents is strongly suggestive of NTM infection. It was also notable that there have been five cases of NTM infection in the Jeju area within a recent year [16].

Treatment regimen with clarithromycin, amikacin, and ciprofloxacin was effective in controlling the infection according to the laboratory results and this patient’s condition. The metallic foreign materials embedded at the site of abscess made the progression of NTM SSTI more severe and rapid than expected. When SSTI is associated with foreign materials, the progression of the clinical course of infection could be unusual and faster. In addition, surgical interventions like incision and drainage in addition to medical treatment is highly recommended for better prognosis when NTM infection is associated with foreign body materials.

Increased public desire to attain and accentuate perceived physical beauty have motivated development of various cosmetic techniques that include lipolysis, acupuncture, and laser therapy. However, the possible risks and side effects of repetitive cosmetic procedures remain unclear. Infection, one of most critical side effects after cosmetic practices, can cause both physical and emotional distress. Bacterial infection goes through a natural course of acute exacerbation and recovery with antibiotics. However, in chronic infection, diagnosis and following treatments tend to be delayed, resulting in permanent sequelae. Therefore, in case of chronic infection, resistant to usual antibiotics regimen, it is helpful to consider the possibility of NTM infection for prompt diagnosis and treatment, and ultimately for a better quality of life in patients.

### Consent

Written informed consent was obtained from the patient for publication of this case report and any accompanying images. A copy of the written consent is available for review by the Editor of this journal.

## Abbreviations

NTM: Nontuberculous mycobacterium; RGM: Rapidly growing mycobacterium; SSTI: Skin and Soft tissue infection; CRP: C-reactive protein; ESR: Erythrocyte sedimentation rate; CT: Computed tomography; AFB: Acid fast bacilli; TB/NTM PCR: *Mycobacterium tuberculosis*/non-tuberculous mycobactera polymerase chain reaction; MFC: *Mycobacterium fortuitum* complex.

## Competing interests

The authors declare that they have no competing interests.

## Authors’ contributions

All authors of this case report made substantial contributions to conception and design. SY and SH drafted the manuscript. KL was involved in the microbiological investigation. SJ performed surgical procedures. All authors revised the manuscript critically, read, and approved the final version.

## Pre-publication history

The pre-publication history for this paper can be accessed here:

http://www.biomedcentral.com/1471-2334/13/479/prepub
